# hiPSC-Derived Epidermal Keratinocytes from Ichthyosis Patients Show Altered Expression of Cornification Markers

**DOI:** 10.3390/ijms22041785

**Published:** 2021-02-11

**Authors:** Dulce Lima Cunha, Amanda Oram, Robert Gruber, Roswitha Plank, Arno Lingenhel, Manoj K. Gupta, Janine Altmüller, Peter Nürnberg, Matthias Schmuth, Johannes Zschocke, Tomo Šarić, Katja M. Eckl, Hans C. Hennies

**Affiliations:** 1 Department of Biological and Geographical Sciences, University of Huddersfield, Queensgate, Huddersfield HD1 3DH, UK; d.cunha@ucl.ac.uk (D.L.C.); amanda.oram@hud.ac.uk (A.O.); roswitha.plank@i-med.ac.at (R.P.); 2 Institute of Human Genetics, Medical University of Innsbruck, Peter-Mayr-Str. 1, 6020 Innsbruck, Austria; arno.lingenhel@i-med.ac.at (A.L.); johannes.zschocke@i-med.ac.at (J.Z.); katja.eckl@edgehill.ac.uk (K.M.E.); 3 Cologne Center for Genomics, University Hospital Cologne, Weyertal 115b, 50931 Cologne, Germany; janine.altmueller@uni-koeln.de (J.A.); nuernberg@uni-koeln.de (P.N.); 4 Department of Dermatology, Venereology and Allergy, Medical University of Innsbruck, Anichstrasse 35, 6020 Innsbruck, Austria; robert.gruber@tirol-kliniken.at (R.G.); Matthias.Schmuth@i-med.ac.at (M.S.); 5 Center for Physiology and Pathophysiology, Institute for Neurophysiology, Medical Faculty, University Hospital Cologne, Robert-Koch-Str. 39, 50931 Cologne, Germany; Manoj.Gupta@joslin.harvard.edu (M.K.G.); tomo.saric@uni-koeln.de (T.Š.); 6 Department of Biology, Edge Hill University, St Helens Road, Ormskirk L39 4QP, UK

**Keywords:** autosomal recessive congenital ichthyosis, cornification, disease modelling, epidermal barrier function, induced pluripotent stem cells, trichothiodystrophy

## Abstract

Inherited ichthyoses represent a large heterogeneous group of skin disorders characterised by impaired epidermal barrier function and disturbed cornification. Current knowledge about disease mechanisms has been uncovered mainly through the use of mouse models or human skin organotypic models. However, most mouse lines suffer from severe epidermal barrier defects causing neonatal death and human keratinocytes have very limited proliferation ability in vitro. Therefore, the development of disease models based on patient derived human induced pluripotent stem cells (hiPSCs) is highly relevant. For this purpose, we have generated hiPSCs from patients with congenital ichthyosis, either non-syndromic autosomal recessive congenital ichthyosis (ARCI) or the ichthyosis syndrome trichothiodystrophy (TTD). hiPSCs were successfully differentiated into basal keratinocyte-like cells (hiPSC-bKs), with high expression of epidermal keratins. In the presence of higher calcium concentrations, terminal differentiation of hiPSC-bKs was induced and markers *KRT1* and *IVL* expressed. TTD1 hiPSC-bKs showed reduced expression of *FLG*, *SPRR2B* and lipoxygenase genes. ARCI hiPSC-bKs showed more severe defects, with downregulation of several cornification genes. The application of hiPSC technology to TTD1 and ARCI demonstrates the successful generation of in vitro models mimicking the disease phenotypes, proving a valuable system both for further molecular investigations and drug development for ichthyosis patients.

## 1. Introduction

Inherited ichthyoses consist of a group of rare disorders of cornification, characterized by skin scaling and hyperkeratosis, and are present as both non-syndromic or syndromic forms [1,2]. Non-syndromic ichthyoses comprise a large spectrum of heterogeneous disorders, including autosomal recessive congenital ichthyosis (ARCI) (OMIM 242300, 242100, 606545, 601277, 242500, 604777, 612281, 615022, 613943, 615023, 602400, 615024, 617320, 617574, 617571). ARCI patients present with generalized scaling of various shapes and colors and different degrees of erythroderma, subcategorized as lamellar ichthyosis (LI), non-bullous congenital ichthyosiform erythroderma, and Harlequin ichthyosis (reviewed in [3]). Variants in 12 different genes have been associated with ARCI so far: *TGM1* (OMIM 190195), *ALOX12B* (OMIM 603741), *ALOXE3* (OMIM 607206), *ABCA12* (OMIM 607800), *CYP4F22* (OMIM 611495), *NIPAL4* (OMIM 609383), *LIPN* (OMIM 613924), *CERS3* (OMIM 615276), *PNPLA1* (OMIM 612121), *CASP14* (OMIM 605848), *SDR9C7* (OMIM 609769) and *SULT2B1* (OMIM 604125).

Trichothiodystrophy (TTD) is a form of syndromic ichthyosis, an autosomal recessive disorder caused by variants in genes encoding subunits of the transcription/repair factor IIH (TFIIH), a multiplex protein that is essential for nucleotide excision repair (NER) and RNA polymerase II-driven transcription [4]. TTD1 (OMIM 601675) is caused specifically by variants in *ERCC2*/*XPD* (OMIM 126340), which encodes a helicase subunit of TFIIH, and typically causes ichthyosis, photosensitivity, hair abnormalities and intellectual and physical disabilities [5].

Ichthyosis patients typically have defects in proteins involved in lipid metabolism and epidermal keratinocyte terminal differentiation and formation of the stratum corneum, causing impaired permeability barrier function [6,7,8,9,10]. In ARCI, most affected gene products were recently linked to a common metabolic pathway required for the processing of epidermal acylceramides, which are essential for the assembly of the epidermal cornified lipid envelope (CLE) and barrier formation (reviewed in [3,11,12]). In contrast, the mechanisms underlying the ichthyosis phenotype in TTD1 are less clear. It is thought that reduced activity of TFIIH alters the transcription levels in late stages of epidermal differentiation. This is supported by the downregulation of late differentiation marker genes *Flg*, encoding filaggrin, and *Sprr2*, coding for a component of the epidermal cornified envelope, in the skin of the TTD mouse model *XPD^R722W^* [13,14,15]. A recent study by Hashimoto et al. reported abnormal lipid metabolism in the skin of *XPD^R722W^* mice, which is related to the reduced expression of liver X receptor (LXR) responsive genes *Abca12* and *Abcg1*, supposedly involved in epidermal glucosylceramide and cholesterol transport [16,17]. In contrast, ARCI mice models show a very severe phenotype with impaired epidermal barrier formation and die soon after birth from transepidermal water loss [18,19,20].

Relevant human model systems are therefore necessary to further understand disease mechanisms and develop causative treatments, which are still non-existent. The generation of human induced pluripotent stem cells (hiPSCs) derived from patient somatic cells became a valuable tool for modelling human diseases, providing a virtually endless source of affected cells for studying disease mechanisms and human development in vitro as well as for drug screening [21]. In the case of cornification disorders, hiPSCs would be of particular advantage, since human primary keratinocytes have very limited proliferative ability in vitro.

The differentiation of pluripotent stem cells into epidermal keratinocytes was first achieved using epidermal stem cells and since then adapted by several groups for obtaining both control and patient-specific basal epidermal keratinocytes from hiPSCs (hiPSC-bKs) [22,23,24,25,26]. However, the propagation of hiPSC-bKs is still rather limited in current approaches and single cell RNA sequencing studies showed that only a small percentage of cells actually resembled primary basal keratinocytes at the molecular level [27], which might hinder the use of these cells for high-throughput goals like establishment of 3D skin equivalents for drug screening.

It is thus important to investigate if patient-derived hiPSC-bKs can be used for the analysis of disease pathogenesis. In this study we report for the first time the establishment of hiPSC-bKs derived from both ARCI patients, with previously unreported causal mutations in *TGM1* and *PNPLA1*, respectively, and a TTD1 patient with mutations in *ERCC2*. Generated hiPSC-bKs displayed morphologies very similar to primary keratinocytes with high expression levels of basal keratins 14 and 5 (K14 and K5). Furthermore, in the presence of high calcium concentrations, hiPSC-derived keratinocytes showed expression of terminal differentiation genes *KRT1* and *IVL*. Disease-specific features were also analyzed with TTD1 hiPSC keratinocytes showing reduced *FLG* and *SPRR2B* expression compared to control keratinocytes. ARCI hiPSC keratinocytes showed disturbance of the terminal differentiation process, with failed expression of several markers of this pathway, including other genes involved in lipid metabolism and mutated in ARCI cases. Our data demonstrate the validity of these cells as an alternative human model system to facilitate the development of causative treatments for ichthyosis patients.

## 2. Results

### 2.1. Generation of Induced Pluripotent Stem Cells (iPSCs)

**Figure 1:**
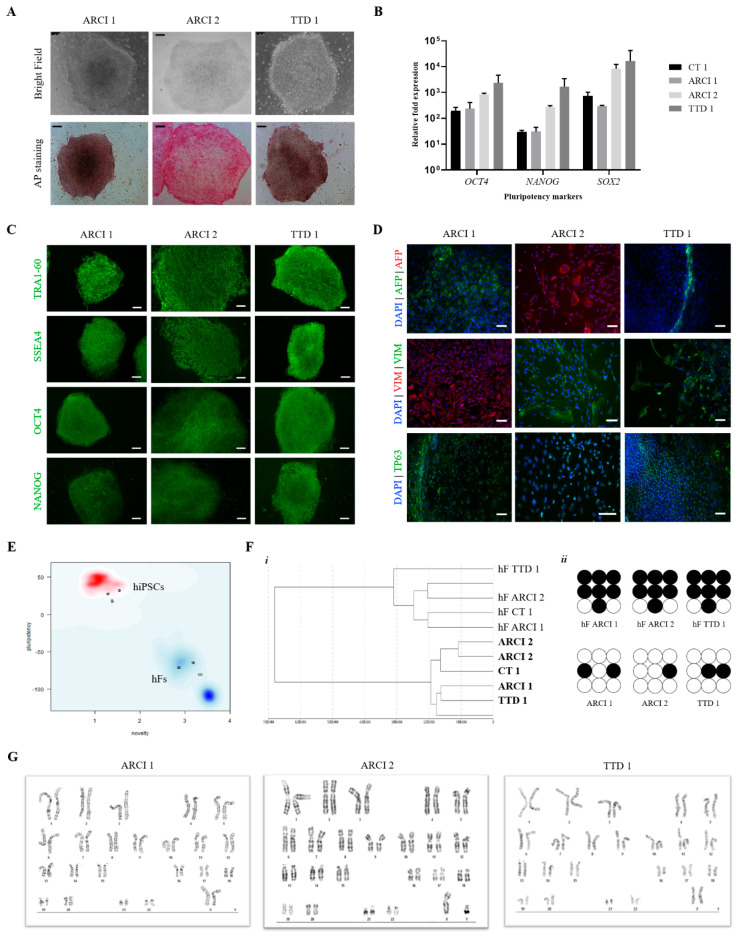
Validation of human induced pluripotent stem cells (hiPSCs) generated from ichthyosis patients. (**A**) Embryonic stem cell (ESC)-like morphology (upper panel) and alkaline phosphatase positive activity (lower panel). Scale bar 250 μm. (**B**) Relative expression of pluripotency transcription factors *OCT4*, *NANOG* and *SOX2* in hiPSCs compared to untransfected human fibroblasts (hFs) by RT-qPCR (*n* = 3). (**C**) Expression of pluripotency markers TRA1-60, SSEA4, OCT4 and NANOG by immunocytochemistry. Expression of these markers was not visible in CF1 mouse embryonic fibroblasts (MEFs). Scale bar 250 μm. (**D**) In vitro differentiation ability of hiPSCs into cells of the 3 germ layers. hiPSCs were spontaneously differentiated and stained positive for specific markers for the 3 germ layers: alpha-fetoprotein (AFP) (endoderm), vimentin (VIM) (mesoderm) and p63 (ectoderm). Scale bar 75 μm. (**E**) Pluritest^®^ analysis of hiPSC whole gene expression showed hiPSCs cluster within the pluripotency “cloud” (red) while respective hFs were allocated close to the blue “cloud” that corresponds to differentiated cells. (**F**) Global DNA methylation status of hiPSCs: (*i*) cluster analysis grouped both cell types in two distinct groups; (*ii*) CpG islands (represented as circles) in *OCT4* promoter region showing demethylated CpGs (white circles) in hiPSCs compared to highly methylated status in hF CpGs (black circles). (**G**) Karyograms of hiPSC lines detected by G-banding analysis.

iPSCs lines were generated by lentiviral transfection of dermal fibroblasts extracted from ichthyosis patients and control donors and expanded in human embryonic stem cell (HES) media in the presence of immortalized CF1 feeder cells. These cells were thoroughly characterized to evaluate their pluripotency profiles, differentiation ability and genomic stability (Figure 1). Colonies presented typical human embryonic stem cell (hESC)-like morphology and stained positive for alkaline phosphatase activity (Figure 1A) and pluripotency markers OCT4, NANOG, SSEA4 and TRA-1-60 (Figure 1C). This was supported by expression analysis using RT-qPCR, which showed that all lines have high expression levels of pluripotency genes *OCT4*, *SOX2* and *NANOG* (Figure 1B). In vitro differentiation ability of hiPSCs was proven by positive expression of markers for the three germ layers after spontaneous differentiation (Figure 1D). Global gene expression analysis using Pluritest™ revealed that generated hiPSC lines cluster within the pool of pluripotent cells (ESCs, hiPSCs) while parental human fibroblasts (hFs) cluster within the differentiated cell pool (Figure 1E). Global methylation profiling showed large differences between parental fibroblasts and generated hiPSC lines, with cluster analysis clearly separating both cell types (Figure 1F-i). When focusing on the methylation status of specific promoters, all hiPSCs showed demethylation of the *OCT4* promoter, which is needed to induce and maintain pluripotency (Figure 1F-ii), while the promoter of *COL1A1*, a fibroblast-specific gene, was clearly methylated following reprogramming (not shown). Lastly, G-banding karyotype analysis showed no chromosomal abnormalities resulting from reprogramming (Figure 1G), and short tandem repeat (STR) marker analysis proved identical genomic profiles between each line and their parental fibroblast line (Table S1).

### 2.2. Genetic Analysis of Patient-Derived hiPSCs

**Figure 2:**
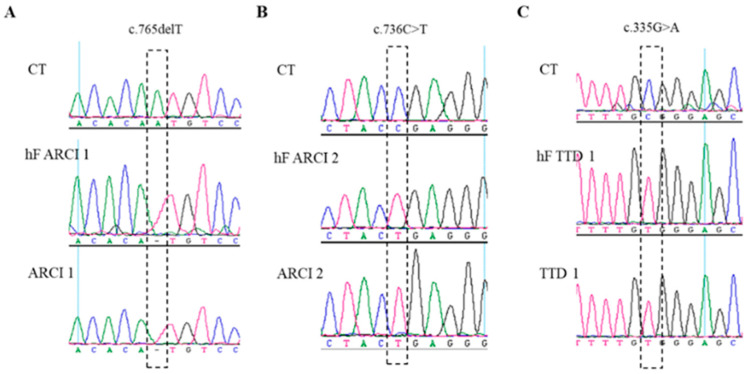
Generated patient-specific hiPSCs maintain the disease-causing variant. DNA was extracted from dermal fibroblasts of a healthy donor (CT), patient-derived dermal fibroblasts (hFs), and hiPSCs (ARCI1, ARCI2 and TTD1, respectively) and analyzed by Sanger sequencing. (**A**) ARCI 1 patient cells showing novel homozygous *TGM1* (NM_000359.3) 1bp deletion c.765delT; (**B**) ARCI 2 patient cells with homozygous nonsense change c.736C>T in exon 5 of *PNPLA1* (NM_001145717); (**C**) TTD1 patient cells showing the homozygous *ERCC2* (NM_000400.4) exon 5 missense change c.335G>A. None of the variants were detected in unaffected control cells (CT).

Disease-causing variants were confirmed in all hiPSC lines generated from patient fibroblasts (Figure 2). hiPSCs from line ARCI1 have a homozygous 1bp deletion in exon 5 of *TGM1*, NM_000359.3:c.765delT, resulting in the frameshift p.(Ile255Metfs*75) (Figure 2A). This variant is considered pathogenic and predicted to lead to transcript degradation by nonsense mediated decay (NMD) by in silico tools. *TGM1* c.765delT is unreported in literature and was not found in ClinVar nor gnomAD repositories.

iPSC line ARCI2 presented the homozygous variant NM_001145717:c.736C>T located in exon 5 of *PNPLA1* that causes a premature stop codon p.(Arg246*), leading to a shortened transcript and likely degradation by NMD (Figure 2B). In silico platforms predicted this variant to be disease-causing and although it is described in dbSNP (rs777268917) and gnomAD with very low frequency (alternate allele frequency 1.2 × 10^−5^), no clinical significance was described prior to this study.

A third hiPSC line was generated from fibroblasts of a TTD1 patient. Parental DNA and hiPSCs carried a missense homozygous variant in exon 5 of *ERCC2*, NM_000400.4:c.335G>A causing the missense change p.(Arg112His) (Figure 2C). This variant has previously been reported in TTD1 patients [28].

### 2.3. Differentiation of hiPSCs into Basal Epidermal Keratinocytes (hiPSC-bKs)

**Figure 3:**
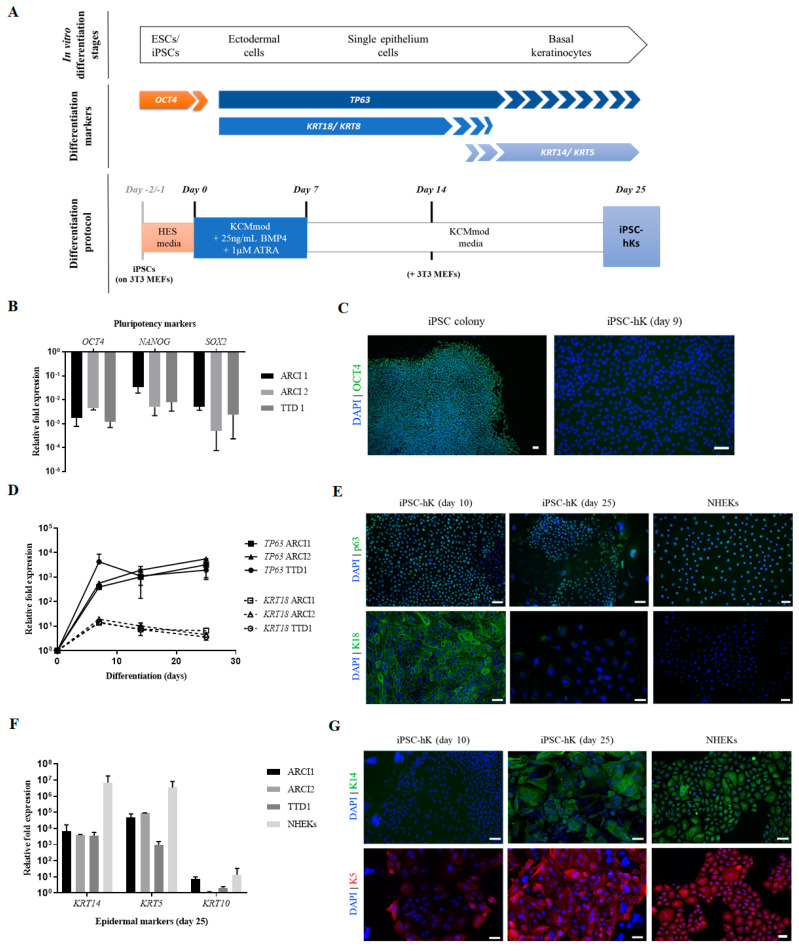
In vitro hiPSC differentiation into hiPSC-derived keratinocytes mimics epidermal morphogenesis. (**A**) Schematic representation of differentiation process and comparison with key stages and markers of epidermal formation. (**B**) Relative expression of pluripotency markers *OCT4*, *NANOG* and *SOX2* after 14 days of differentiation (minimum *n* = 2). (**C**) OCT4 protein was undetected by immunocytochemistry approx. 9 days post-differentiation compared to positive control (undifferentiated hiPSC colony). Scale bar 50 μm. (**D**) Relative expression of early ectodermal markers *TP63* and *KRT18* throughout the differentiation process; *KRT18* levels at day 25 are significantly reduced compared to day 7 (*p* < 0.001) (minimum *n* = 2). (**E**) Representative immunocytochemistry of differentiating hiPSC-bKs at days 10 and day 25 showing expression of p63 and keratin 18 (K18). Normal primary keratinocytes (NHEKs) were used as positive controls. Scale bar 50 μm. (**F**) Relative expression of basal keratinocytes-specific *KRT14* and *KRT5* in hiPSC-bKs after 25 days of differentiation (minimum *n* = 2). (**G**) Representative immunocytochemistry of hiPSC-bKs after 10 and 25 days and NHEKs, showing expression of basal keratinocyte-specific keratins 14 (K14) and 5 (K5). Scale bar 50 μm.

hiPSC differentiation into epidermal keratinocytes was performed as outlined in Figure 3A with hiPSC lines ARCI1, ARCI2 and TTD1, and the previously characterized hiPSC line WT2 (also named UCLi017-A) generated from dermal hFs of a skin healthy control person [29]. Briefly, when confluent, hiPSC were plated onto 3T3 J2 feeders in very low density and allowed to grow in HES media for 24–48h. Induction of epidermal fate started by changing the media to keratinocyte culture medium (KCM) with FGF7/KGF (KCMmod) supplemented with epidermal inducers BMP-4 and all-trans retinoic acid (ATRA) for 7 days. After that point, cells were further cultured in KCMmod without epidermal inducers. Although the differentiation process has been previously described in defined conditions, we found that the presence of 3T3 J2 feeder cells improved differentiation efficiency and resulted in hiPSC-bKs with better morphology and survival rates. Similarly, the addition of FGF7/KGF to the media instead of EGF, a typical component of KCM, produced hiPSC-bKs with better morphology and slightly improved the expression of basal keratinocytes-specific keratins (not shown).

Morphology of differentiating hiPSCs was visibly different by day 7, where exit from pluripotent state was confirmed by marked downregulation of *OCT4* (10^3^-fold), *NANOG* (up to 100-fold) and *SOX2* (up to 10^4^-fold decrease) in patient-derived hiPSC-bKs (Figure 3B). Accordingly, no OCT4 protein was detected by immunocytochemistry in differentiating cells (Figure 3C).

At the same time point, early ectodermal and epithelial markers *TP63* and *KRT18* were highly upregulated (10^3^- and 10-fold, respectively), proving ectodermal and single epithelial commitment, respectively (Figure 3D). These markers are also detected in all cells by day 10, as shown by immunocytochemistry analysis (Figure 3E). From day 7 onwards, *TP63* expression seems to stabilize while *KRT18* expression slowly decreases (Figure 3D). The downregulation of *KRT18* seems more evident from day 14, at the same time as expression of basal keratins *KRT14* and *KRT5* starts to greatly increase, being highly upregulated at day 25/30 (Figure 3F). This could indicate that the signal for inducing stratified epithelium in our system might surge around day 14. Some K5 staining, but not K14, is already visible in a few cells as early as day 10. Expression of both keratins continues to increase until day 25/30, at which stage both can be detected in the great majority of hiPSC-bKs (Figure 3G).

### 2.4. Expansion and Terminal Differentiation of hiPSC-bKs

After 25 days of differentiation, hiPSC-bKs resembled primary basal keratinocytes in both morphology and expression of specific keratins *KRT14* and *KRT5* (Figure 3F,G). At this point, ARCI1, TTD1 and WT2 cells were split onto feeder-free conditions in collagen I-coated dishes and grown in CnT-07 media for further expansion. Unfortunately, further differentiation into epidermal keratinocytes did not succeed for ARCI2 hiPSCs. Similarly to primary basal keratinocytes, hiPSC-bKs also seem to be difficult to expand in vitro due to low survival and/or proliferation rates. However, the addition of 10 µM of Y-27632, a commonly used Rho kinase inhibitor, to the expansion media dramatically improved hiPSC-bK morphology and proliferation ability: eight days after splitting, cells cultured in the presence of Y-27632 maintained their keratinocyte-like appearance. Alternatively, in the absence of this compound, proliferation of hiPSC-bK seemed stalled and a majority of cells with a fibroblast-like morphology developed (Figure S1).

**Figure 4:**
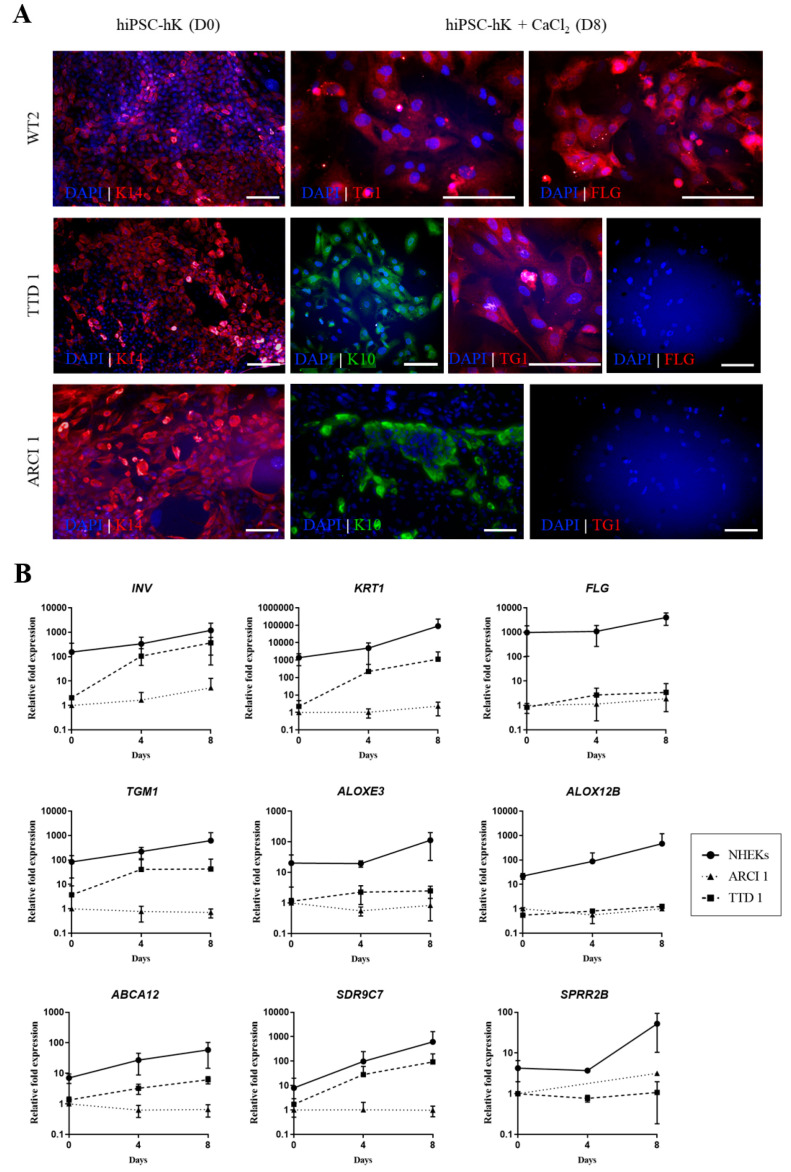
Calcium-induced terminal differentiation of patient-derived hiPSC-bKs. (**A**) Immunocytochemistry of basal keratinocytes marker keratin 14 (K14) and cornification proteins keratin 10 (K10), transglutaminase 1 (TG1) and filaggrin (FLG) in terminally differentiating control WT2 and ichthyosis patient TTD1 and ARCI1 hiPSC-derived keratinocytes. Scale bar 100 µm. (**B**) Relative expression of several genes involved in the cornification process in both ARCI1 and TTD1. Calcium-exposed NHEKs were used as controls. All values were normalized to day 0 ARCI1 hiPSC-bKs with internal gene *GAPDH* used as reference.

Increase of calcium concentrations in culture media induces the terminal differentiation of primary human keratinocytes (hKs) in vitro [30]. Morphology of hiPSC-derived keratinocytes changed drastically after 8 days of calcium exposure but variability between both patient lines was visible, with better differentiation ability seen in TTD1 cells compared to ARCI1 (Figure S2). Accordingly, terminal differentiation marker keratin 10 (K10) was detected in the majority of TTD1 and ARCI1 keratinocytes after calcium exposure, proving these cells have the ability to terminally differentiate (Figure 4A). As expected, differentiated ARCI1 hiPSC-derived keratinocytes did not show *TGM1* expression in contrast to TTD1 and the control WT2. On the other hand, TTD1 showed a downregulation of the terminal differentiation marker filaggrin (Figure 4A).

Gene expression analysis showed further defects in ARCI1 terminal differentiation. Although some upregulation of markers *KRT10* and *IVL* was detected using RT-qPCR, proving the terminal differentiation process is triggered in vitro, expression of most cornification genes was profoundly decreased, as compared to both TTD1 and normal human epidermal keratinocytes (NHEKs), suggesting the cornification process is affected due to the loss of TG1 activity (Figure 4B). TTD1 cells showed increased expression of *KRT1, IVL* and *TGM1*. However, TTD1 hiPSC-bKs also presented a disturbed terminal differentiation process with downregulation of *FLG* and *SPRR2B* compared to NHEKs, which is similarly seen in TTD1 patients and TTD mouse models. Surprisingly, we also saw reduced expression of *ALOXE3* and especially *ALOX12B*, which has not been previously reported in TTD1 skin (Figure 4B). These results demonstrate that hiPSC-bKs generated from hFs of ichthyosis patients can terminally differentiate in vitro and recapitulate molecular defects characteristic of the disease.

## 3. Discussion

This study presents an hiPSC-based approach for in vitro modelling of ichthyoses. Several hiPSC lines were generated by reprogramming of dermal fibroblasts from patients diagnosed with congenital ichthyosis, namely ARCI and TTD1. All hiPSC lines demonstrated typical features of pluripotent cells that clearly distinguished them from their respective fibroblast origins. hiPSC lines used for differentiating into skin cells also did not present karyotype abnormalities and patient-specific lines retained the respective causal variants.

The hiPSCs were successfully differentiated into epidermal keratinocytes using BMP-4 and ATRA for the initial induction into epithelial fate, complemented with KGF/FGF7 during the differentiation process. After 25 days, hiPSC-derived keratinocytes revealed a basal epidermal signature, proven by high expression levels of basal keratinocyte specific genes *KRT14*/*KRT5*, while pluripotency related markers *OCT4, NANOG* and *SOX2* were undetected. The process of differentiation of hiPSCs into basal keratinocytes also resembled the developmental process of epidermal formation, mimicking the expression patterns of key progenitor markers *TP63* and *KRT18* in this process.

Both EGF and KGF/FGF7 have been reported to favor interfollicular epidermal fate by promoting basal keratinocytes proliferation during skin morphogenesis [31,32,33]. However, EGF-exposed cultures were reported to have higher presence of non-keratinocytes skin cells, pointing to a more specific action of KGF/FGF7 [34]. In our system, the use of KGF/FGF7 instead of EGF clearly improved the efficiency of the process, resulting in higher expression levels of *KRT14/KRT5* as well as improved morphology of hiPSC-bKs.

After differentiation, hiPSC-bKs were expanded in feeder-free conditions for further analysis. However, and much like primary keratinocytes, hiPSC-bKs also seemed to have reduced proliferation ability in vitro, which may limit their downstream use. We show here that treatment with Rho kinase inhibitor Y-27632 significantly improves the morphology and proliferation ability of hiPSC-bKs. Accordingly, this compound has been shown to improve survival and proliferation of both primary keratinocytes as well as hiPSC-derived retinal pigmented cells [35,36]. Importantly, Y-27632 might have inhibitory effects on keratinocyte terminal differentiation [37]; therefore, it was only added during hiPSC-bK expansion. Senescence of primary keratinocytes treated with Y-27632 was reported after ~70 days in culture [35]; Y-27632 supplementation does not represent a source of immortalized keratinocytes.

In order to validate these cells for modelling of keratinisation disorders, their ability to terminally differentiate in vitro is essential. Hence, in response to high calcium concentrations, hiPSC-derived keratinocytes were shown to express early differentiation marker genes *KRT1* and *KRT10* and terminal differentiation marker *IVL*, while presenting defects in this process that are consistent with the respective disease phenotypes. TTD1 hiPSC keratinocytes showed increased *KRT1*, *TGM1* and *IVL* expression after calcium exposure but markedly reduced expression of *FLG* compared to control WT2 hiPSC-derived keratinocytes and NHEKs. This is consistent with the literature, where reports compare the skin phenotype of TTD1 patients to ichthyosis vulgaris patients, who have loss-of-function *FLG* mutations [13,14,38]. In addition, we found a marked downregulation of *SPRR2B*, which has been identified as decreased in the skin of TTD1 mice model *XPD^R722W^* [14,39]. Recently, it was reported that these animals also show reduced expression of *Abca12* [16]; in our system, TTD1 hiPSC-keratinocytes expressed *ABCA12,* although at lower levels as compared to NHEKs. Furthermore, we observed a proper upregulation of *SDR9C7*; SDR9C7 was very recently identified as responsible for the dehydrogenation of acylceramides, which occurs just before covalent crosslinking to proteins of the cell envelope involving TG1 [40]. We found a reduced expression of epidermal LOX genes, particularly *ALOX12B*, in our TTD1 cells. These genes code for two lipoxygenases, eLOX3 and 12*R*-LOX, responsible for the oxygenation of acylceramides in the later stages of CLE formation. These results further corroborate the evidence towards a common pathomechanism in TTD1 and ARCI skin phenotype, although a direct role of TFIIH or the XPD subunit in the establishment of the epidermal permeability barrier has not yet been uncovered.

ARCI1 hiPSCs were derived from a patient with a homozygous loss-of-function mutation in *TGM1*. These cells were successfully differentiated into basal hK-like cells but terminal differentiation showed striking abnormalities in both morphology and gene expression. Terminally differentiated ARCI1 hiPSC-keratinocytes showed no expression of *TGM1* in contrast to WT2 and TTD1, as expected due to their genotype, but also failed to express the majority of genes involved in cornification and epidermal barrier formation, except for a moderate increase in *KRT1* and *IVL* levels. These results pointed to a broader deregulation of this pathway consistent with clinical findings, although TG1 is a known player of the very last steps in epidermal barrier formation.

Recently, microarray analyses of skin biopsies from ARCI patients with *TGM1* variants revealed upregulation of several other ARCI-related and cornification genes, suggested as a compensatory mechanism to increase acylceramide production and consequently improve the barrier function [41]. In our in vitro system, the majority of genes related to this pathway were downregulated in ARCI1 iPSC-derived keratinocytes, possibly due to differences between fresh samples and cultured cells. Accordingly, expression data from cultured *TGM1* KD as well as TG1 deficient patient hKs showed downregulation of most cornification genes compared to unaffected hKs, except for *KRT1,* which is comparable to control lines (data unpublished). Interestingly, Zhang et al. found the most significant increase in ichthyosis-related genes in patients with *TGM1* missense variants and ongoing retinoid treatment, while single patients without treatment and with a homozygous splice site mutation, respectively, did not show these clear upregulations. Further studies will be necessary and further patients will have to be analyzed to determine if the variant type or current treatments might have an influence on gene expression and the dysregulation of keratinocyte differentiation.

Finally, it should be mentioned that the defects of ARCI1 hiPSC-bKs could also be (partially) caused by the hiPSC line differentiation ability itself; it has been shown that these properties of hiPSCs can differ greatly between lines, regardless if they were generated from healthy or affected donors [42,43]. Indeed, in our system, differentiation of hiPSCs into hiPSC-bKs and especially the expansion of hiPSC-bKs, were more successful in TTD1 hiPSCs than in ARCI1. On the other hand, ARCI1 hiPSC-bKs exhibited morphology and basal keratin expression comparable to TTD1 hiPSC-bKs and control hiPSC-bKs, suggesting these defects are restricted to the later stages of differentiation. Importantly, this is consistent with the patient phenotype, where ARCI patients carrying *TGM1* variants present with more severe skin features than TTD patients.

This is the first report of hiPSCs established from ichthyosis patients where we successfully differentiated these cells into basal hK-like cells and further pushed them into a 2D terminal differentiation process. Our terminally differentiated hiPSC-derived keratinocytes from both ARCI and TTD1 patients showed different degrees of disturbed cornification, consistent with ichthyoses primary keratinocytes in vitro, proving they can mimic and be used to further elucidate the molecular pathomechanisms underlying this heterogeneous group of disorders. Future studies including organotypic models will be needed to further characterize functional changes associated with ichthyosis, including epidermal barrier analysis and protein and lipid profiles. Finally, these cells can also provide a valuable in vitro platform of patient-derived cells to be used in drug development. In ARCI, where advancements in protein replacement strategies have recently been reported [44,45], hiPSC-based 2D or 3D models would be a particularly important tool. Patient-derived hiPSCs can be a long-term source for a variety of different cells and, thus, significantly contribute to the development of complex skin disease models, eventually containing patient-derived melanocytes and immune cells and ultimately facilitating the translation of therapeutic approaches into clinical studies.

## 4. Materials and Methods

### 4.1. Ethical Approvals

The study was approved by the Ethics Committees of the Medical University of Innsbruck, Austria (UN4501, 12 Dec 2011), and the University of Cologne, Germany (11-274, 1 Dec 2011), as well as by the School Research Ethics and Integrity Committee at the University of Huddersfield, UK (SAS-SREIC 4.1.19-11, 4 Jan 2019, and SAS-SREIC 23.4.20-3A, 24 June 2020). All human primary cell lines used in this project were obtained after written informed consent from patients (or parents in case of underage patients) and healthy donors in accordance with Declaration of Helsinki protocols.

### 4.2. Donor and Patient Description

Patient ARCI 1, is a female, 3 months old at the time of the biopsy collection and of Middle Eastern origin. The patient later presented with coarse and generalized brown-colored scales, mild erythroderma and with a collodion membrane at birth, being diagnosed with LI. Patient ARCI 2, male, was 12 years old at the time of biopsy collection, presented with moderate LI and mild erythroderma. Patient TTD 1, female, deceased at the age of 4 years, of central European origin, presented with congenital ichthyosis on the upper body and had brittle sulphur-deficient hair and recurrent infections, being diagnosed with photosensitive TTD. Control skin samples were obtained from healthy donors from the Medical University of Innsbruck or purchased from Genoskin (France).

### 4.3. Variant Analysis

Pathogenic variants of ichthyosis patients were identified previously using Sanger sequencing or gene panel sequence analysis. For this study, genomic DNA was extracted from patient fibroblasts and hiPSCs with Genomic DNA Minikit (Invitrogen, Carlsbad, CA, USA), following the kit instructions. Primers for *TGM1* exon 5, *PNPLA1* exon 5 and *ERCC2* exon 5 were designed with Primer 3 [46] (available upon request). PCR was performed using standard protocols. Products were purified and prepared for sequencing reaction with BigDye^®^ Terminator v1.1 Kit (Applied Biosystems, Foster City, CA, USA) and run in a Sequence Analyser 3130XL (Applied Biosystems, Foster City, CA, USA). Sequences were analyzed with SeqmanPro software (DNASTAR Inc., Madison, WI, USA). Variant pathogenicity prediction was performed by in silico tools MutationTaster [47], SIFT [48], Polyphen-2 [49] and Human Splicing Finder 3.0 [50]. Variant novelty was accessed by searching ClinVar (https://www.ncbi.nlm.nih.gov/clinvar/) and GnomAD v2.1.1 (https://gnomad.broadinstitute.org/).

### 4.4. Induced Pluripotent Stem Cells (iPSC) Generation

hSTEMCCA and helper plasmids were isolated from transformed *Escherichia coli* with Purelink HiPure Maxiprep Kit (Invitrogen, Carlsbad, CA, USA). Production of lentivirus was based in Tiscornia et al. [51]. Plasmid DNA were added to packaging HEK293FT cells (Invitrogen, Carlsbad, CA, USA) with Calcium Phosphate Kit (Sigma-Aldrich, Saint Louis, MO, USA). After harvesting, supernatant-containing viral particles was concentrated with LentiX Concentrator (Clontech Laboratories, Mountain View, CA, USA) and virus was titrated using p24 ELISA assay (Clontech Laboratories, Mountain View, CA, USA). Patient hFs were infected with lentivirus with MOI = 125 following established protocols [52]. After infection, cells were passaged onto CF1 mouse embryonic fibroblasts (MEFs; ScienCell Research Laboratories, Carlsbad, CA, USA) and fed daily with HES media for 3–4 weeks until hiPSC colonies appeared.

### 4.5. Cell Culturing

Primary keratinocytes and fibroblasts used in this study were isolated from skin punch biopsies or plastic surgery surplus as described by Eckl et al. [53]. Primary human keratinocytes were cultured in medium KCM [54] on a 3T3 J2 feeder cell layer or, when required, in feeder-free conditions with defined KGM (Lonza, Basel, Switzerland). Human dermal fibroblasts were grown in DMEM containing 10% FCS and supplemented with 2 mM glutamine, 100 IU/mL penicillin, and 100 µg/mL streptomycin (all from Gibco, Life Technologies, Carlsbad, CA, USA).

hiPSCs were cultured in 0.1% gelatin-coated dishes (Sigma-Aldrich, Saint Louis, MO, USA) in the presence of Mitomycin C-treated CF1 MEFs (ScienCell Research Laboratories, Carlsbad, CA, USA) and fed daily with HES media (DMEM/F12 with Glutamax, 20% Knockout Serum replacement, 10 mM NEAA, 0.1 mM β-mercaptoethanol, 100 IU/mL penicillin, and 100 µg/mL streptomycin, all from Gibco, Life Technologies, Carlsbad, CA, USA) supplemented with 50 ng/mL of basic FGF (Peprotech, Rocky Hill, NJ, USA). All cells were maintained at 37 °C, 95% humidity, 5% CO_2_.

hiPSC-derived embryoid bodies (EBs) were formed by plating small hiPSC clumps on low attachment culture plates with AggreWell EB Formation Medium (StemCell Technologies, Vancouver, Canada) supplemented with 10 µM Y-27632 (Sigma-Aldrich, Saint Louis, MO, USA). EBs were grown for 7–10 days, then plated onto gelatin-coated dishes for adherence and fed with DMEM, 20% FBS, 1% Penicillin/Streptomycin (Gibco, Life Technologies, Carlsbad, CA, USA) for cell proliferation for another 15 days.

### 4.6. Differentiation of hiPSCs into Keratinocytes (iPSC-hKs)

Differentiation of hiPSCs into basal keratinocytes-like cells (iPSC-hKs) is summarized in Figure 3A: hiPSCs were split manually when 80% confluent onto 0.1% porcine gelatin-coated dishes (Sigma Aldrich, Saint Louis, MO, USA) seeded with 3T3 J2 MEFs in HES media for 24–48h. On day 0, the medium was changed to KCM supplemented with 10 ng/mL KGF/FGF7 (Peprotech, Rocky Hill, NJ, USA) (KCMmod) plus known epidermal-lineage inducers ATRA (1 µM, Sigma-Aldrich, Saint Louis, MO, USA) and BMP-4 (25 ng/mL, Peprotech, Rocky Hill, NJ, USA). The medium was changed every other day for 7 days. Afterwards, inducers were removed and cells were fed with KCMmod every other day until day 25.

### 4.7. Expansion and Terminal Differentiation of hiPSC-bKs

On day 25 of differentiation, hiPSC-bKs were collected with Accumax (Gibco, Life Technologies, Carlsbad, CA, USA) and plated onto collagen I-coated dishes (100 µg/mL, Advanced Biomatrix) in CnT-07 media (CellnTec, Bern, Switzerland) supplemented with 10 µM Y-27632 (Sigma-Aldrich, Saint Louis, MO, USA). A rapid attachment step of approx. 20 min was performed to allow attachment of only hiPSC-bKs. Cells were fed every other day until confluent when they were used for downstream analysis. For assessing terminal differentiation ability of hiPSC-bKs and control hKs, cells were changed to CnT-PR-D media (CellnTec, Bern, Switzerland) without Y-27632 24h before confluency and, the next day, 1.2 mM CaCl_2_ (Sigma-Aldrich, Saint Louis, MO, USA) was added to the differentiation media. Samples were taken for analysis after 4 and 8 days.

### 4.8. Alkaline Phosphatase Staining

Alkaline phosphatase activity was detected following the instructions of AP Staining Kit II (Stemgent, Cambridge, MA, USA).

### 4.9. RT-qPCR

RNA was extracted from hKs, hFs, hiPSCs and hiPSC-bKs using PureLink RNA Mini Kit (Invitrogen, Carlsbad, CA, USA), following the kit’s instructions. RNA concentrations and integrity were measured using Nanodrop 2000 (Thermo Fisher Scientific, San Diego, CA, USA). 500 ng RNA was used for cDNA synthesis using High Capacity RNA-to-cDNA Kit (Applied Biosystems, Foster City, CA, USA) in a total volume of 20 µL. Reactions were incubated at 37°C for 1h and 95°C for 5 min. cDNA was then diluted 1:10 and 2.5 µL of each diluted cDNA sample was added to 2.5 µL of DNAse-free H_2_O and 5 µL of TaqMan^®^ Fast Universal PCR Master Mix, making a total volume of 10 µL per reaction in pre-designed Custom Taqman^®^ Array Fast Plates containing previously validated Taqman^®^ assays (Table S2) (all from Applied Biosystems, Foster City, CA, USA). Samples were run in a Step One Plus PCR System (Thermo Fisher Scientific, San Diego, CA, USA) in duplicates or triplicates with the following cycle conditions: 95°C for 20s, followed by 40 cycles of 95°C for 1s and 60°C for 20s. Expression levels were determined using the ΔΔC_t_ method and 18S RNA and *GAPDH* were used independently as reference genes. A minimum of two independent biological replicates were analysed in each experiment.

### 4.10. Immunofluorescence

Methanol-fixed hiPSCs, hiPSC-bKs and primary hKs were used for immunofluorescence staining following standard protocols, with antibodies detailed in Table S3. Fluorescence was analyzed with a Leica LMD6000 microscope (Leica, Wetzlar, Germany).

### 4.11. Karyotype Analysis

hiPSC karyotypes were routinely analysed by G-banding karyotyping. A minimum of 20 metaphases were analyzed for each iPSC line at each time point.

### 4.12. Short Tandem Repeat (STR) Analysis

Genomic stability was confirmed by amplification of 6 different STR markers following standard protocols and analysis on a Sequence Analyser 3130XL (Applied Biosystems, Foster City, CA, USA). Fragment sizes were analyzed with PeakScanner 2 software (Applied Biosystems, Foster City, CA, USA).

### 4.13. Whole Gene Expression Profiling

RNA from feeder-free iPSCs and hFs were extracted using PureLink RNA Mini Kit (Invitrogen, Carlsbad, CA, USA) and ran in an Illumina HT-12v3 array (Illumina, San Diego, CA, USA). Resulting raw files were uploaded and analyzed in Pluritest™ website [55].

### 4.14. Global DNA Methylation Array

DNA methylation array was performed after extraction of DNA from hiPSC lines and hFs using an Infinium HumanMethylation450 BeadChip v1.1 (Illumina, San Diego, CA, USA). Data was analyzed with GenomeStudio 2.0 software (Illumina, San Diego, CA, USA).

### 4.15. Statistical Analysis

Statistical analysis for RT-qPCR results was performed using Multiple *t*-test in GraphPad Prism 8.0.2 (GraphPad Software Inc., San Diego, CA, USA). All graphs show data of at least two independent experiments with duplicates. Samples were determined statistically significant when *p* < 0.05.

## Data Availability

Not available.

## Supplementary Materials

Supplementary materials can be found at https://www.mdpi.com/1422-0067/22/4/1785/s1. Figure S1. Effect of Y-27632/Rho kinase inhibitor (RI) on ARCI1 and TTD1 hiPSC-bKs proliferation and morphology; Figure S2. Calcium-induced terminal differentiation of patient-derived hiPSC-bKs; Table S1. Short tandem repeats (STR) fragment sizes of patient hiPSCs and their respective parental fibroblast (hF) lines; Table S2. Taqman^®^ assays used in this study; Table S3. Primary antibodies used in this study.

## Abbreviations

ARCI	Autosomal recessive congenital ichthyosis
ATRA	All-trans retinoid acid
CLE	Cornified lipid envelope
HES	Human embryonic stem cell
hF	Human dermal fibroblast
hK	Human epidermal keratinocyte
hiPSC	Human induced pluripotent stem cell
hiPSC-bK	hiPSC-derived basal keratinocyte
KCM	Keratinocyte culture medium
LI	Lamellar ichthyosis
MEF	Mouse embryonic fibroblast
NHEK	Normal human epidermal keratinocytes
STR	Short tandem repeat
TTD	Trichothiodystrophy
