# Supplementary material for: hiPSC-Derived Epidermal Keratinocytes from Ichthyosis Patients Show Altered Expression of Cornification Markers
Source: Int J Mol Sci. 2021 Feb 11;22(4):1785. doi: 10.3390/ijms22041785 (PMC7916893; doi:10.3390/ijms22041785)
Supplement: Supplementary file 1 [file ijms-22-01785-s001.pdf]

International Journal of *Molecular Sciences*

MDPI

Article

hiPSC-derived epidermal keratinocytes from ichthyosis patients show altered expression of cornification markers

Dulce Lima Cunha[1,2,3,†], Amanda Oram[1], Robert Gruber[4], Roswitha Plank[1,2], Arno Lingenhel[2], Manoj K. Gupta[5,‡], Janine Altmüller[3], Peter Nürnberg[3], Matthias Schmuth[4], Johannes Zschocke[2], Tomo Šarić[5], Katja M. Eckl[2,6], Hans C. Hennies[1,2,3,*]

[1] Department of Biological and Geographical Sciences, University of Huddersfield, Queensgate, Huddersfield HD1 3DH, United Kingdom; d.cunha@ucl.ac.uk (DLC); amanda.oram@hud.ac.uk (AO); roswitha.plank@i-med.ac.at (R.P.); h.c.hennies@hud.ac.uk (HCH)
[2] Institute of Human Genetics, Medical University of Innsbruck, Peter-Mayr-Str. 1, 6020 Innsbruck, Austria; d.cunha@ucl.ac.uk (DLC); roswitha.plank@i-med.ac.at (RP); arno.lingenhel@i-med.ac.at (AL); johannes.zschocke@i-med.ac.at (JZ); katja.eckl@edgehill.ac.uk (KML); h.c.hennies@hud.ac.uk (HCH)
[3] Cologne Center for Genomics, University Hospital Cologne, Weyertal 115b, 50931Cologne, Germany; d.cunha@ucl.ac.uk (DLC); janine.altmueller@uni-koeln.de (JA); nuernberg@uni-koeln.de (PN); h.c.hennies@hud.ac.uk (HCH)
[4] Department of Dermatology, Venereology and Allergy, Medical University of Innsbruck, Anichstrasse 35, 6020 Innsbruck, Austria; robert.gruber@tirol-kliniken.at (RG); Matthias.Schmuth@i-med.ac.at (MS)
[5] Center for Physiology and Pathophysiology, Institute for Neurophysiology, Medical Faculty, University Hospital Cologne, Robert-Koch-Str. 39, 50931 Cologne, Germany; Manoj.Gupta@joslin.harvard.edu (MKG); tomo.saric@uni-koeln.de (TŠ)
[6] Department of Biology, Edge Hill University, St Helens Road, Ormskirk L39 4QP, United Kingdom; katja.eckl@edgehill.ac.uk (KML)
† Current address: UCL Institute of Ophthalmology, 11-43 Bath Street, London EC1V 9EL, UK
‡ Current address: Takeda Pharmaceuticals, Cambridge, MA 02139, USA
* Correspondance: h.c.hennies@hud.ac.uk; phone +44-1484-473014

SUPPLEMENTARY MATERIALS

Supplementary figures

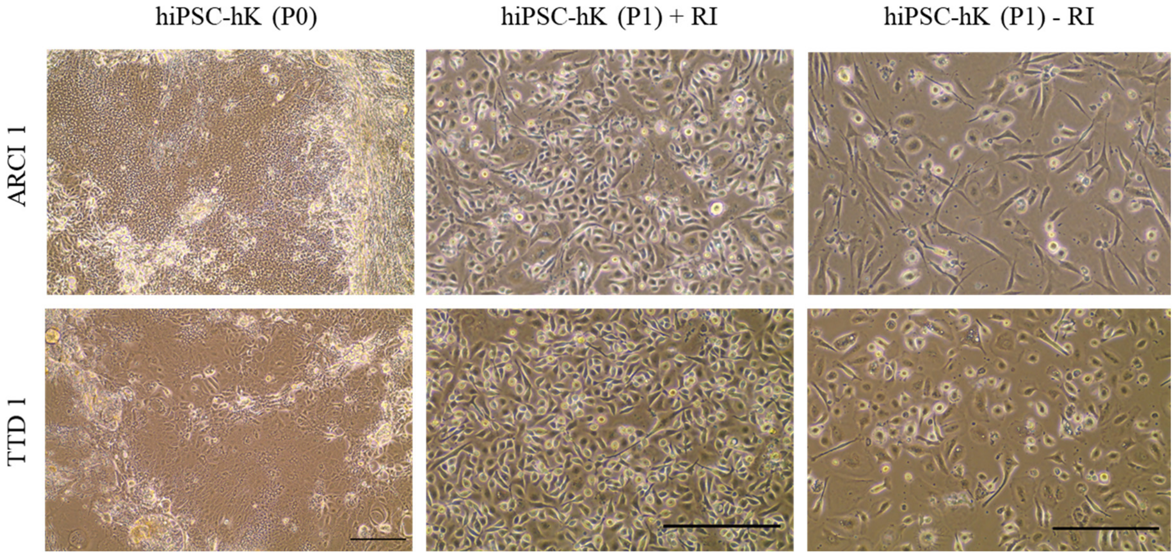

Figure S1. Effect of Y-27632/Rho kinase inhibitor (RI) on ARCI1 and TTD1 hiPSC-bKs proliferation and morphology. Passage 0 (P0) hiPSC-bKs were split and passage 1 (P1) cells were cultured in media with 10 μM Y-27632 (+RI) and without Y-27632 (-RI) for 8 days. Scale bar 100 μm.

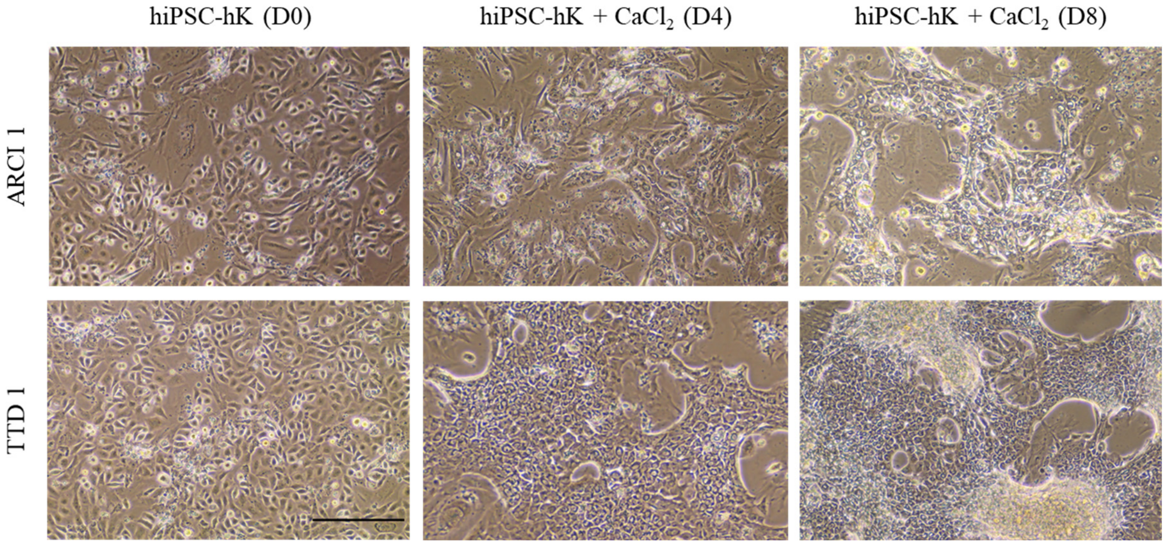

Figure S2. Calcium-induced terminal differentiation of patient-derived hiPSC-bKs. Morphology of ARCI1 and TTD1 hiPSC-bKs is shown on day 0 (D0) and after 4 (D4) and 8 (D8) days of 1.2 mM $CaCl_2$ exposure. Scale bar 100 μm.

Supplementary tables

Table S1. Short tandem repeat (STR) fragment sizes of patient hiPSCs and their respective parental fibroblast (hF) lines. Values represent the base pair length of each allele fragment sizes for each of the 6 markers.

	D1S466	GAAT1A4	D16S2621	D18S976	D4S2632	D6S1045
**ARCI1 (hF)**	162/172	150/154	254/254	174/174	118/118	126/126
**ARCI1 (hiPSC)**	162/172	150/154	254/254	174/174	118/118	126/126
**ARCI2 (hF)**	160/166	136/154	246/250	172/184	118/144	122/126
**ARCI2 (hiPSC)**	160/166	136/154	246/250	172/184	118/144	122/126
**TTD1 (hF)**	156/160	136/154	238/250	178/180	138/138	118/130
**TTD1 (hiPSC)**	156/160	136/154	238/250	178/180	138/138	118/130

Table S2. Taqman® assays used in this study.

Name	Assay	Fragment size
18S RNA	Hs99999901_s1	187
ABCA12	Hs00292421_m1	77
ALOX12B	Hs00153961_m1	73
ALOXE3	Hs00222134_m1	112
FLG	Hs00856927_g1	137
GAPDH	Hs99999905_m1	112
IVL	Hs00846307_s1	148
KRT1	Hs00196158_m1	86
KRT14	Hs00265033_m1	64
KRT18	Hs02827483_g1	64
KRT5	Hs00361185_m1	133
NANOG	Hs04399610_g1	101
OCT4	Hs00999632_g1	77
SDR9C7	Hs00541011_m1	64
SOX2	Hs01053049_s1	91
TGM1	Hs01070310_m1	99
TP63	Hs00978343_m1	85

Table S3. Primary antibodies used in this study.

Target	Dilution	Company details
Alpha-feto protein (AFP)	1:100	#Sc51506, Santa Cruz
K10	1:150	Abcam #ab53124
K14	1:400	#MAB3232, Millipore
K18	1:400	#MAB3234, Millipore
K5	1:500	#ab24647, Abcam
Nanog	1:100	#Sc33759, Santa Cruz
Oct4	1:100	#Sc5279, Santa Cruz
p63	1:50	#Sc8431, Santa Cruz
p63	1:200	#619002, Biolegend
SSEA 1	1:50	#MAB4301, Chemicon
SSEA 4	1:50	#MAB4303, Chemicon
TG1	1:100	#Sc-166467, Santa Cruz
TRA-1-60	1:50	#MAB4360, Chemicon
TRA-1-81	1:50	#MAB4381, Chemicon
Vimentin	1:250	#Sc7557, Santa Cruz
